# Roads to isolation: Similar genomic history patterns in two species of freshwater crabs with contrasting environmental tolerances and range sizes

**DOI:** 10.1002/ece3.4017

**Published:** 2018-04-15

**Authors:** Ywee Chieh Tay, Daniel Jia Jun Ng, Jun Bin Loo, Danwei Huang, Yixiong Cai, Darren Chong Jinn Yeo, Rudolf Meier

**Affiliations:** ^1^ Department of Biological Sciences National University of Singapore Singapore City Singapore; ^2^ Tropical Marine Science Institute National University of Singapore Singapore City Singapore; ^3^ National Biodiversity Centre National Parks Board Singapore City Singapore; ^4^ School of Chemical and Life Sciences Singapore Polytechnic Singapore City Singapore; ^5^ Lee Kong Chian Natural History Museum National University of Singapore Singapore City Singapore

**Keywords:** COI, depauperate genetic diversity, habitat fragmentation, island endemic, population genomics, RAD‐seq

## Abstract

Freshwater species often show high levels of endemism and risk of extinction owing to their limited dispersal abilities. This is exemplified by the stenotopic freshwater crab, *Johora singaporensis* which is one of the world's 100 most threatened species, and currently inhabits less than 0.01 km^2^ of five low order hill streams within the highly urbanized island city‐state of Singapore. We compared populations of *J. singaporensis* with that of the non‐threatened, widespread, abundant, and eurytopic freshwater crab, *Parathelphusa maculata*, and found surprisingly high congruence between their population genomic histories. Based on 2,617 and 2,470 genome‐wide SNPs mined via the double‐digest restriction‐associated DNA sequencing method for ~90 individuals of *J. singaporensis* and *P. maculata,* respectively, the populations are strongly isolated (*F*_ST_ = 0.146–0.371), have low genetic diversity for both species (also for COI), and show signatures of recent genetic bottlenecks. The most genetically isolated populations for both species are separated from other populations by one of the oldest roads in Singapore. These results suggest that anthropogenic developments may have impacted stream‐dependent species in a uniform manner, regardless of ubiquity, habitat preference, or dispersal modes of the species. While signs of inbreeding were not detected for the critically endangered species, the genetic distinctiveness and low diversity of the populations call for genetic rescue and connecting corridors between the remaining fragments of the natural habitat.

## 1 INTRODUCTION

Freshwater‐dependent species are often poor dispersers (Cumberlidge, 2008; Keikhosravi, Fratini, & Schubart, 2015; Sterling, Reed, Noonan, & Warren, 2012; Whiterod, Zukowski, Asmus, Gilligan, & Miller, 2016) because they tend to be heavily dependent on connected freshwater bodies. This is particularly so for species that inhabit transient running water habitats (Chester, Miller, Valenzuela, Wickson, & Robson, 2015; Shurin, Cottenie, & Hillebrand, 2009). In species with inherently low dispersal abilities such as fully aquatic or water‐dependent fish or amphibians, the physical isolation of freshwater bodies during periods of low rainfall or changes in hydrology often result in a high rate of population divergence (Keikhosravi et al., 2015; Sterling et al., 2012; Whiterod et al., 2016), and endemism (Collen et al., 2014; Strayer & Dudgeon, 2010). Such diversifying forces are often intensified via anthropogenic impacts such as habitat fragmentation and degradation. Prolonged isolation of populations, especially small populations, may lead to inbreeding, reduced genetic fitness, and greater vulnerability to disturbances that increase the risk of extirpation (Reed, 2008; Wilcox & Murphy, 1985). Island populations of freshwater species, with the exception of catadromous species with an oceanic dispersal phase (Hymanson, Wang, & Sasaki, 1999), are likely subject to inbreeding and reduced genetic fitness due to their isolation from larger, mainland populations (Boff, Soro, Paxton, & Alves‐dos‐Santos, 2014; Furlan et al., 2012; Jensen et al., 2013). Island freshwater endemics are therefore one of the most vulnerable groups (Howard et al., 2015; Kier et al., 2009) and suffer from some of the highest extinction rates (Frankham, 1998).

**Figure 1:**
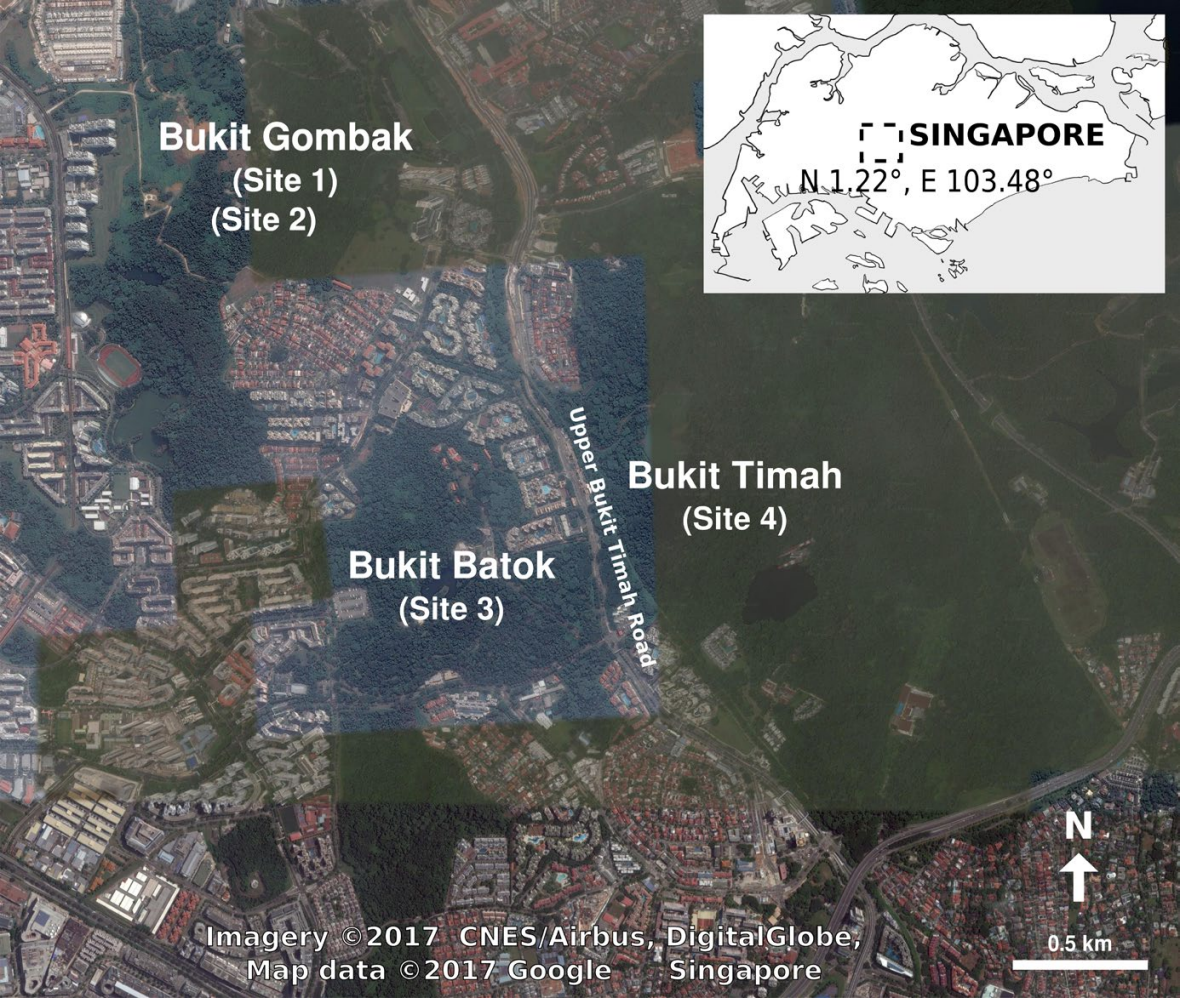
Approximate sampling localities (exact stream localities omitted to prevent poaching), with the study area indicated in the rectangle in the inset map of Singapore. Satellite imagery downloaded from Google Maps at https://maps.google.com.sg/

Despite catastrophic extinctions of 34%–87% in forest specialists over a span of just 183 years (Brook, Sodhi, & Ng, 2003), the main island of Singapore (<720 km^2^ in size) hosts a handful of endemic freshwater species including one of the 100 globally most threatened species, the potamid freshwater crab *Johora singaporensis* Ng, 1986 (Baillie & Butcher, 2012). The genus *Johora* consists of 15 species of primary freshwater crabs found in highland areas of the Malay Peninsula from southern Thailand to Singapore (Ng, 1988; Yeo, Shih, Meier, & Ng, 2007), and *J. singaporensis* is a stenotopic species that requires relatively fast‐flowing, well‐oxygenated, and pristine streams (Chua, Ng, Zeng, & Yeo, 2015). This species is currently listed as Critically Endangered on the IUCN Red List (Esser, Cumberlidge, & Yeo, 2008), and is only known from small hill streams within the Bukit Timah, Bukit Batok, and Bukit Gombak areas in Singapore (Figure 1; Ng, 1986, 1990; Yeo, Tan, & Ng, 2008; Ng, Yeo, Sivasothi, & Ng, 2014; Ng, Sivasothi, Cai, Davison, & Yeo, 2015; Li, Lim, & Cai, 2015) although historically, it may have been found throughout most of Singapore's hill streams. Unfortunately, land use changes over the past two centuries have drastically altered these habitats, reducing the species’ distribution to a small fraction of its historical range (Ng, McGowan, et al., 2015). Indeed, *J. singaporensis* appears to have been extirpated from its type locality in recent years (Ng et al., 2014). It is estimated that only a few hundred reproductive mature individuals remain in the wild (Ng, McGowan, et al., 2015), otherwise not much is known about the biology of this critically endangered species.

Here, we assess whether island isolation and anthropogenic impacts have influenced the genomic history of the critically endangered freshwater crab *J. singaporensis* and provide population genomic insight that can help with its conservation. A fine‐scale population genomic analysis was performed for the stenotopic *J. singaporensis*, and the widespread sympatric and often syntopic freshwater crab species, *Parathelphusa maculata* De Man, 1879; comparing environmental tolerance and range size. This widespread species has numerous “mainland” populations in Peninsular Malaysia and Sumatra, and is ubiquitous across the lowland freshwater streams of Singapore island (Ng, 1988). As it is unlikely for individuals of *P. maculata* to be able to disperse across a saltwater barrier to Singapore from other mainland populations, any island isolation effects in Singapore, if present, might be expected to affect both species equally and lead to a shared overall genetic history. Based on recent observations by the second author, three populations of *J. singaporensis* currently inhabit the same streams as *P. maculata*, while the remaining two populations share adjacent forest habitats. At present, the crab populations in different forest patches appear to be geographically isolated. Here, we assess the population genomic histories in both species using genome‐wide SNPs and mitochondrial cytochrome *c* oxidase I (COI) data.

## 2 MATERIALS AND METHODS

Sampling, bench work, and data analyses were performed in the same manner for both crab species unless stated otherwise.

### 2.1 Site locations and sample collection

We sampled *J. singaporensis* from four streams located just outside the Central Catchment Nature Reserve of Singapore. The sites—two in Bukit Gombak (Sites 1 & 2), one in Bukit Batok (Site 3) and one in Bukit Timah Nature Reserve (Site 4)—were selected based on the known distribution of *J. singaporensis* (Ng et al., 2014; Ng, Sivasothi, et al., 2015). These sites cover four of five known populations of the species (Figure 1; precise localities withheld due to poaching risks). *Parathelphusa maculata*, which co‐occurs with *J. singaporensis* in three of these streams, was also collected, with additional sampling of a fifth stream in Bukit Timah Nature Reserve that lacked *J. singaporensis*. In all cases, 20–25 individuals of each species were sampled.

Fully intact subadult or adult individuals were located at night (1900–2200 h) and captured by hand. Given the threatened status of *J. singaporensis*, a nonlethal method that does not significantly harm the crabs was employed; each crab was released after carefully removing one ambulatory leg, which was immediately stored in absolute ethanol on ice. The tissues were brought back to the laboratory and kept at −30°C until DNA extraction.

### 2.2 DNA extractions, COI genotyping, ddRAD‐Seq library preparation and Illumina sequencing

DNA was extracted from 20 to 26 samples per site using the phenol–chloroform method. Briefly, leg tissues were rinsed with autoclaved water, air‐dried, pulverized and then incubated at 55°C for 18 h in 800 μl CTAB buffer with 400 μg of proteinase K. The lysate was mixed well and washed with two rounds of 500 μl phenol–chloroform–isoamyl alcohol (25:24:1) followed by precipitation with 100% ethanol and a 70% ethanol wash. The final purified DNA pellet was eluted in ~40 μl molecular grade water, and stored at −20°C for further analyses.

Cytochrome *c* oxidase I (COI) genotyping was performed using the primer pairs LoboF1 and LoboR1 (Lobo et al., 2013), and HCO2198 and LCO1490 (Folmer, Black, Hoeh, Lutz, & Vrijenhoek, 1994) for *J. singaporensis* and *P. maculata*, respectively. PCRs were performed in 25 μl reactions containing 2 μl of 10–40 ×  diluted DNA extract, 1× reaction buffer, 0.8 mmol/L total dNTPs, 0.4 μmol/L each primer and 1U BioReady rTaq polymerase. Reactions for *J. singaporensis* required the addition of 2 μg bovine serum albumin. Cycling parameters were 94°C (1 min), 5 cycles of 94°C (30 s), 45°C (1 min 30 s) and 72°C (1 min), 30 cycles of 94°C (30 s), 54°C (1 min 30 s) and 72°C (1 min), followed by a final extension of 72°C (5 min) for *J. singaporensis*; 94°C (1 min), 34 cycles of 94°C (45 s), 48°C (45 s) and 72°C (1 min 30 s), followed by a final extension of 72°C (3 min) for *P. maculata*. Successfully amplified products were purified using SureClean^™^ (Bioline) following the manufacturer's instructions, and sequenced using BIGDYE^™^ v. 3.1 (Applied Biosystems Inc.) on an ABI Avant 3130xl Genetic Analyzer.

Prior to ddRAD‐Seq library preparation, the DNA extracts were quantified using a NanoDrop 1000 and assessed for quality on a 1% agarose gel. Of the extracted samples, 94 individuals were selected for ddRAD‐Seq such that each sampling locality was represented by 23 to 24 individuals for each species. The ddRAD‐Seq libraries were prepared as in Tay et al. (2016), with modifications as follows: (1) magnetic bead‐PEG buffer suspension:DNA ratios were narrowed (0.81×, 0.95×), (2) PCR cycles were reduced from 18 to 16, and (3)an additional round of size selection on the pooled product was performed at the end to ensure a clean product. For pooling purposes, individuals from each sampling location had a unique Illumina Index, and each sample within those pools had a unique 5 bp barcode (Table S1). Two Illumina HiSeq 2500 100 bp paired‐end sequencing runs were performed per species (94 samples per lane). After data demultiplexing from the first run, sample pooling and sequencing were repeated with sample load adjustment to target an even coverage per sample. Each run was spiked with 5%–10% genomic DNA libraries. Sequencing was outsourced to the Genomics Institute of Singapore.

For the following analyses, default parameters were used unless stated otherwise.

### 2.3 Population genetic analyses—mitochondrial

COI sequence chromatograms were edited, assembled, translated to check for stop codons, aligned, and trimmed to 589 bp and 680 bp in Geneious v. 9.1.6 for *P. maculata* and *J. singaporensis*, respectively. Haplotype (*h*_d_) and nucleotide (π) diversities were calculated using DnaSP v. 5 (Librado & Rozas, 2009). Representative sequences for each haplotype were deposited with Genbank (Accession numbers MG010138–MG010329). Statistical parsimony haplotype networks were inferred using the TCS method (Clement, Snell, Walke, Posada, & Crandall, 2002) in POPART (Leigh & Bryant, 2015). Also included in the haplotype network for *P. maculata* are six COI sequences (Klaus et al., 2013; Poettinger & Schubart, 2014) retrieved from Genbank, but data for the 105 specimens were trimmed to the shortest of 568 bp. Population demographic changes for each species were assessed using Bayesian Skyline Plots (BSP) using BEAST v. 1.7 (Drummond, Suchard, Xie, & Rambaut, 2012) under the GTR nucleotide substitution model as recommended by jModelTest 2 (Darriba, Taboada, Doallo, & Posada, 2012; Guindon & Gascuel, 2003). Three runs were performed for each species, with tip dates set to zero, under the uncorrelated relaxed clock with a mutation rate of 2.33% Myr^−1^ (previously calibrated for Jamaican sesarmids; Schubart, Diesel, & Hedges, 1998) for 2 × 10^8^ MCMC iterations and discarding 10% as burn‐in. Runs were considered converged when effective sample sizes were >200 in TRACER v 1.5 (Rambaut & Drummond, 2007). Further analyses that could have yielded additional information such as population demographic changes at the individual population, and population genetic differentiation estimates were abandoned after preliminary results indicated that the results would not likely be meaningful due to the low genetic variability in the COI data for each species.

### 2.4 Population genetic analyses—single nucleotide polymorphisms from ddRAD‐Seq

Raw Illumina sequence quality was assessed using FastQC v. 0.11.2 (Andrews, 2012). Only data from Read 1 were used in the analyses. Loci were assembled, and SNPs called using individual modules of the de novo pipeline in STACKS v. 1.42 (Catchen, Hohenlohe, Bassham, Amores, & Cresko, 2013) as follows: (1) Reads were demultiplexed using *procees_radtags* in which low‐quality reads below Phred score 20 were discarded, reads with uncalled bases removed, and reads trimmed to 83 and 87 bp for *J. singaporensis* and *P. maculata* data sets, respectively, as Phred scores dropped below 20 beyond those bases (Figure S1). One of the sequencing runs for *P. maculata* was overwhelmed by one particular sample due to unequal pooling; hence, only data from one lane were used for subsequent analyses. For *J. singaporensis*, data from both runs were pooled but 1.2 × 10^8^ reads were randomly subsampled via jackknifing (script in Supplementary data, “jackknife.py”) to obtain a comparable number of reads to *P. maculata*, and all subsequent demultiplexed reads were filtered for potential chimeras using a custom bash script (Tay et al., 2016); (2) A sensitivity analysis of assembly of putative loci into “stacks” was performed using *ustacks* under different parameter combinations. Parameter settings tested include minimum “stack” depths (−m) of 5 and 10, and maximum number of mismatches between “stacks” (−M) of 1, 2, 4, 8. Secondary reads were not retained; (3) Loci catalogs were assembled per species from these “stacks” in *cstacks*, with mismatches allowed (−n) following the mismatch thresholds for −M; (4) Loci were identified by matching “stacks” to the catalogs using *sstacks;* (5) SNPs were called using *populations* with minimum “stack” depths (−m) of 10, 20, 30, where at least 80% of individuals per population were required to be present at a locus (−r), and a minor allele frequency (MAF) (−a) filter such that at least three copies of an allele are present. For the *J. singaporensis* data set, two samples were removed due to low coverage from subsequent analyses. To check for possible contamination despite the data filtering, consensus sequences from the assembled catalogs of loci were matched against the entire NCBI nucleotide database and just the bacterial database alone. BLAST matches of *e*‐value ≤1 × 10^−14^ were not significant. Quick assessments for effects of different SNP‐calling parameter sets on the genetic clustering patterns across the samples were made for data sets with mismatch thresholds of 2 and 8 (with some of the highest and lowest SNP calls; Figure S2), via discriminant analysis of principal components (DAPC) (Jombart, Devillard, & Balloux, 2010) using the R (R Development Core Team, 2009) package Adegenet v. 2.0.2 (Jombart, 2008) (Figure S3). As these runs produced similar genetic clustering patterns across all parameter combinations, only data sets with some of the highest SNP calls are presented here for clarity (mismatch thresholds of 2 bp, minimum stack and final locus depths of 5 and 10, respectively). Conversion of file formats from STACKS output files was made using PGDSpider v. 2.0.5.1 (Lischer & Excoffier, 2012). Possible outlier loci were identified using Bayescan v. 2.1 (Foll & Gaggiotti, 2008) with a burn‐in of 5 × 10^4^ followed by a total of 1 × 10^6^ iterations. Loci were determined to be under balancing or purifying selection when alpha values were zero or negative (Foll & Gaggiotti, 2008; Pilot et al., 2014), and under diversifying selection when positive, at a false discovery rate (FDR) of 0.05 (Figure S4). Sequences identified to be under diversifying selection and deviating from Hardy–Weinberg equilibrium were removed.

Final datasets were thus filtered for quality, outlier loci, and samples with low coverage. Genetic diversity indices were calculated in STACKS and also using the *divBasic* option in the R package diveRsity (Keenan, McGinnity, Cross, Crozier, & Prodöhl, 2013). Genetic structure across the samples was assessed using the following three methods: (1) Bayesian clustering analyses under models of admixture and correlated allele frequencies in STRUCTURE (Falush, Stephens, & Pritchard, 2003; Hubisz, Falush, Stephens, & Pritchard, 2009; Pritchard, Stephens, & Donnelly, 2000) were performed with a burn‐in of 1 × 10^5^ followed by 3 × 10^5^ iterations for data collection. Ten replicate runs of each model of one to six genetic clusters (*K*) were automated using StrAuto (Chhatre & Emerson, 2017), with the addition of a few extra short runs at *K* = 1 such that starting seed values for each of the actual runs were different. The model with highest likelihood for genetic clustering was assessed using STRUCTURE HARVESTER (Earl & vonHoldt, 2012; Evanno, Regnaut, & Goudet, 2005), and the ten independent runs for this model were matched using the *Greedy* algorithm in CLUMPP v. 1.1 (Jakobsson & Rosenberg, 2007). Resulting barplots were constructed using Distruct v. 1.1 (Rosenberg, 2003). (2) DAPC (Jombart et al., 2010), was performed using Adegenet for genetic clustering that does not rely on any population genetic model and is thus free from assumptions of HWE or linkage disequilibrium. (3) Pairwise population differentiation statistics (*G*′_ST_ (Hedrick, 2005), *D*_Jost_ (Jost, 2008), *F*_ST_ (Weir & Cockerham, 1984)) were calculated using the *fastDivPart* option with 100 bootstrap replicates (across loci) in diveRsity. Signatures of recent genetic bottlenecks were estimated using the heterozygosity excess method in BOTTLENECK (Cornuet & Luikart, 1996)—populations at mutation‐drift equilibrium are expected to have a roughly equal probability for heterozygosity excess or deficit, whereas genetic bottleneck events can cause a greater reduction in allelic diversity (e.g., via the loss of rare alleles) than heterozygosity. Two random subsets of 350 SNP loci were generated and genetic bottlenecks were estimated with 1 × 10^4^ replications under the Infinite Allele Model of mutation which is the recommended model for SNP data (Cornuet & Luikart, 1996). Significant deviations from mutation‐drift equilibrium were assessed using a one‐tailed Wilcoxon signed rank test. Contemporary effective population sizes were estimated for each sampling locality using a bias‐corrected version of the linkage disequilibrium method (LDNe; Waples & Do, 2008) implemented in NeEstimator v. 2.01 (Do et al., 2014). Lowest allele frequency was defined at 0.05 to exclude single copy alleles, as rare alleles tend to bias linkage disequilibrium estimates upward (Waples & Do, 2010).

## 3 RESULTS

### 3.1 Population genetic analyses—mitochondrial data

**Figure 2:**
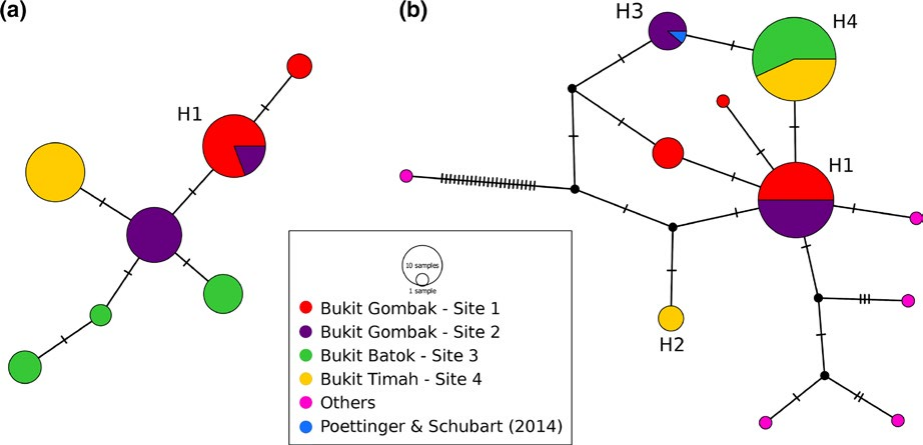
Statistical parsimony haplotype networks for (a) *Johora singaporensis* and (b) *Parathelphusa maculata*. Each circle represents one COI haplotype, and the sizes of the circles are proportionate to the number of individuals with that haplotype (scale inset in legend). Each hatch mark represents one mutational step between haplotypes. Sampling locations are color‐coded as indicated in legend. Note that the haplotype network depicted here for *P. maculata* is based on the trimmed dataset of 568 bp—in the full 589 bp Singapore‐only dataset, samples from Sites 3 and 4 in H4 are two different haplotypes

Genetic diversity estimates based on COI sequence data for both species were low, with only seven haplotypes found for either species across the four sampling localities in Singapore (*h*_JSIN_ = 0.00–0.64, *h*_PMAC_ = 0.00–0.44; Table 1). Overall haplotype diversity of *J. singaporensis* was slightly higher than the diversity of *P. maculata* (*h*_JSIN_ = 0.80 vs. *h*_*PMAC*_ = 0.76; Table 1). Haplotypes were not very divergent from each other with a maximum of only 1 bp mismatch between the nearest haplotypes in *J. singaporensis* (Figure 2a). In *P. maculata*, the maximum number of mismatches between nearest haplotypes was 3 bp before trimming to 568 bp (Figure 2b). Samples collected outside of Singapore were generally more distant, while the one sample previously collected from Bukit Gombak in Singapore by Poettinger and Schubart (2014) clustered with individuals collected from Site 2 in Bukit Gombak (H3, Figure 2b). Strong genetic structure was found among all sampling localities: Only Sites 1 and 2 in Bukit Gombak shared common haplotypes for both species (H1, Figure 2). BSP plots suggest population contractions approximately 1,500 to 2,500 years before present in both species (Figure S5). Due to the observed low levels of genetic variation in the COI datasets, population‐level analyses such as population demographic changes may be less meaningful; hence, only general trends instead of absolute values were inferred.

**Table 1:** Population diversity statistics. Based on COI sequence data as calculated in DnaSP for both crab species, where *N*_COI_ = number of individuals, *N*_H_ = number of haplotypes, *h*_*d*_ = haplotype diversity, π_COI_ = nucleotide diversity. Based on SNP data as calculated in STACKS and diveRsity, where *N*_SNP_ = number of individuals, Private = number private alleles, π_SNP_ = nucleotide diversity, *H*_O_ = observed heterozygosity, *H*_E_ = expected heterozygosity, *F*_IS_ = inbreeding coefficient, N_e_ = effective population size (estimated in NeEstimator)

	COI				ddRAD‐Seq						
	*N*_COI_	*N*_H_	*h*_d_ ± *SD*	π_COI_ ± *SD*	*N*_SNP_	Private	π_SNP_	*H*_O_	*H*_E_	*F*_IS_	*N*_e_ (95% CI)
*Johora singaporensis*											
Bukit Gombak Site 1	25	2	0.28 ± 0.10	0.00042 ± 0.00015	22	111	0.0008	0.33	0.25	−0.33	−23.2 (∞)
Bukit Gombak Site 2	25	2	0.33 ± 0.10	0.00050 ± 0.00015	22	60	0.0007	0.31	0.22	−0.37	−21.3 (∞)
Bukit Batok Site 3	20	3	0.64 ± 0.06	0.00231 ± 0.00017	21	162	0.0008	0.33	0.25	−0.3	−24.8 (∞)
Bukit Timah Site 4	23	1	—	—	22	249	0.0008	0.33	0.24	−0.34	−19.7 (∞)
*Parathelphusa maculata*											
Bukit Gombak Site 1	25	3	0.44 ± 0.10	0.00078 ± 0.00019	22	188	0.0013	0.17	0.16	−0.04	229.8 (201.8, 266.4)
Bukit Gombak Site 2	26	2	0.44 ± 0.07	0.00226 ± 0.00037	23	112	0.0014	0.19	0.18	−0.07	29.2 (28.6, 29.8)
Bukit Batok Site 3	25	1	—	—	22	83	0.0012	0.16	0.15	−0.06	53.2 (51.0, 55.5)
Bukit Timah Site 4	23	2	0.30 ± 0.11	0.00204 ± 0.00071	22	413	0.0012	0.15	0.14	−0.07	−427.9 (∞)

### 3.2 Population genetic analyses—single nucleotide polymorphisms from ddRAD‐Seq

91.9 ± 3.0% and 78.8 ± 7.4% of demultiplexed reads for *J. singaporensis* and *P. maculata,* respectively, were retained after quality filtering in STACKS. For both species, 1.2–3.2 × 10^6^ of these ddRAD‐Seq reads were retained per sample after further filtering for potential chimeric reads (96.4%–99.7%), apart from the *P. maculata* individual which was overrepresented (Figure S6). With minimum final “stack” depths of 10 and the MAF filter applied, 1,219–2,645 SNPs were called (Figure S2). After removing 28 and zero outlier loci potentially under diversifying selection (Figure S4), analyses with data sets containing 2,617 and 2,470 SNPs are presented for *J. singaporensis* and *P. maculata,* respectively. Nucleotide diversity of the nuclear SNP data was low in both species, as was seen in the mitochondrial data (π‐nuclear_PMAC_ = 0.0012–0.0014, π‐nuclear_JSIN_ = 0.0007–0.0008; Table 1).

**Figure 3:**
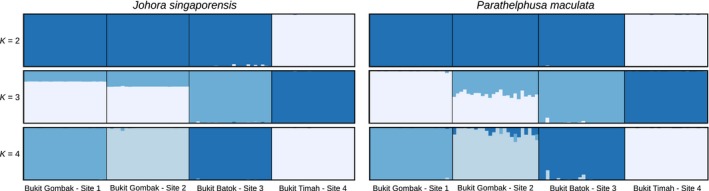
STRUCTURE barplots depicting the proportion of membership (*y*‐axes) of each individual (*x*‐axes) to the predicted genetic clusters when *K* = 2 to *K* = 4, calculated over ten iterations. Different genetic clusters are represented by different shades of blue. (Left) *Johora singaporensis* and (right) *Parathelphusa maculata*

**Figure 4:**
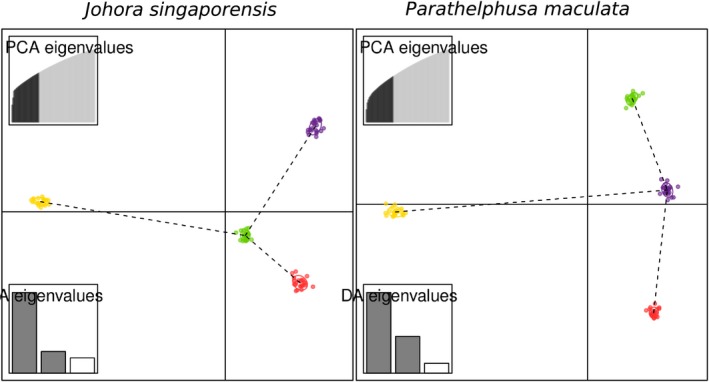
DAPC plots where sampling locations are color‐coded as in Figure 2. (Left) *Johora singaporensis* and (right) *Parathelphusa maculata*

All three methods of population genetic structure showed similar patterns of highly differentiated populations for both species, with the population at Bukit Timah (Site 4) being most distinct from the other three populations. While the Evanno method (Evanno et al., 2005) identified two main genetic clusters via the Bayesian model in STRUCTURE for both species (Figure S7b,d), the plateaus of the mean likelihood and second peak in delta *K* at *K* = 4 (Figure S7a–c) suggest the presence of substructure within these two main genetic clusters. STRUCTURE barplots at *K* = 2 clearly show the population at Bukit Timah (Site 4) to be the most distinct from the other three populations, but also distinct as four populations at *K* = 4 (Figure 3). DAPC plots are free from assumptions and hence possible violations of assumptions of population genetic models, such as when effective population sizes are small, but also showed the same patterns, as the three populations at Bukit Gombak (Sites 1 and 2) and Bukit Batok (Site 3) are much closer to one another on the first discriminant function (Figure 4). Pairwise W&C *F*_ST_ were all high and significant (*F*_ST JSIN_ = 0.181–0.324, *F*_ST PMAC_ = 0.146–0.371) with none of the 95% confidence intervals containing zero (Table 2), and these *F*_ST_ values were ~1.2–2.5× higher for pairwise comparisons with the population at Bukit Timah (Site 4).

**Table 2:** Pairwise W&C *F*_ST_ comparisons based on SNP data. *F*_ST_ and 95% confidence intervals for *Johora singaporensis* and *Parathelphusa maculata* are below and above the diagonal, respectively. All significant pairwise *F*_ST_ values are indicated in bold

	Bukit Gombak, Site 1	Bukit Gombak, Site 2	Bukit Batok, Site 3	Bukit Timah, Site 4
Bukit Gombak, Site 1		**0.146** (0.137, 0.162)	**0.284** (0.275, 0.300)	**0.371** (0.363, 0.383)
Bukit Gombak, Site 2	**0.194** (0.187, 0.203)		**0.165** (0.149, 0.179)	**0.335** (0.327, 0.344)
Bukit Batok, Site 3	**0.181** (0.174, 0.191)	**0.195** (0.189, 0.205)		**0.364** (0.355, 0.374)
Bukit Timah, Site 4	**0.291** (0.284, 0.298)	**0.324** (0.318, 0.332)	**0.290** (0.283, 0.298)	

Mode‐shifts in allele frequencies observed for both species suggest recent genetic bottlenecks, except for the *P. maculata* population at Bukit Gombak Site 2 which showed an L‐shaped distribution. However, highly significant departures in heterozygosity from mutation‐drift equilibrium were detected for all sampling localities for both crab species (*p* = .000, except at Bukit Timah for *P. maculata p* = .00384), which are strong indications of genetic bottlenecks. Significant heterozygote excess was observed in *J. singaporensis*, where *H*_O_ = 0.31–0.33 while *H*_E_ = 0.22–0.25 (Table 1). Estimates of contemporary effective population sizes (Ne) of *P. maculata* populations were mostly low (29.2 to 229.8) with narrow 95% confidence intervals; however, the present data were inconclusive for Bukit Timah and all populations of *J. singaporensis*, which had negative lower, and infinite upper bounds (Table 1).

## 4 DISCUSSION

This is the first comparison of the population genomics of a critically endangered stenotopic island endemic freshwater species with a co‐occurring species that is more abundant and widespread (i.e., freshwater crabs occur together in the same forest streams). The much larger population size of *P. maculata* led us to hypothesize that anthropogenic impacts are likely to have had greater effects on the fine‐scale genetic history of *J. singaporensis*. However, our data show that these two crab species with vastly different population demographics nevertheless share similar population genetic histories with a pattern of low genetic diversity, genetic isolation between populations, and a recent genetic bottleneck. Yet, the surprising lack of inbreeding signatures in both species suggests that population demographic changes were recent, contrary to expectations of lower genetic diversity and more isolated populations in the stenotopic and endemic *J. singaporensis*. Interestingly, we found that the patterns of population isolation in both species were consistent with the chronological order of landscape changes that accompanied urbanisation in the island‐state of Singapore.

Strong genetic structure is typical for many freshwater‐dependent species with limited overland dispersal abilities (Keikhosravi et al., 2015; Sterling et al., 2012; Whiterod et al., 2016). Urbanization is likely to modify and in many cases strengthen this structure by reducing population genetic diversity and intensifying population divergence (Lourenço, Álvarez, Wang, & Velo‐Antón, 2017; Munshi‐South, Zolnik, & Harris, 2016). The small geographic scale of the present study (<1–2.5 km), might lead to the expectation that these crabs are able to move between drainage system during periods of heavy rain and flooding of the terrain between streams. However, we found no evidence for such dispersal. Instead, the genetic differentiation in both species of freshwater crabs was more consistent with the hypothesis that population isolation had been driven by landscape changes, specifically urbanization, during the development of Singapore since its founding almost 200 years ago. We traced the earliest possible records of urban development (roads, buildings, and plantations) from historical maps with sufficient resolution in the Bukit Gombak and Bukit Timah areas (maps downloaded from the National Archives Singapore, or NAS, in 2016, at http://www.nas.gov.sg/archivesonline/maps_building_plans/ for 1839, 1939, 1945 and 1969), and found that the chronology of urban development correlated well with the population genetic differentiation signals from the genome‐wide SNP data. Strong genetic structure is observed for both species between the Bukit Timah population (Site 4) and those at Bukit Gombak and Bukit Batok (Sites 1–3) which could be explained by the isolation of Site 4 when Upper Bukit Timah Road was constructed. This is one of the oldest major roads in Singapore and was already present in some form by 1839 as a north–south connection through the island, and possibly earlier with the founding of Singapore as a British port‐settlement in 1819 (Turnbull, 1989). By 1939, Bukit Batok and Bukit Gombak were dominated by belukar (secondary forest vegetation) and rubber plantations (as documented in the historical maps from NAS), which may have posed a mild barrier to freshwater crab dispersal.

Arguably, population genetic divergence may, to a lesser extent, also be influenced by the crabs’ natural history. *Jorora singaporensis* is more semi‐terrestrial than *P. maculata* which may have reduced dispersal via stream networks. This may explain why all its populations on the west side of the Upper Bukit Timah Road (Sites 1–3) have similar genetic divergence (pairwise *F*_ST_ values = 0.181–0.195; Table 2). In contrast, *P. maculata* is an aquatic stream‐living species that generally occurs fully submerged in deeper water, and is hence more likely to disperse by movement within connected lowland stream networks (Ng, 1988). Hence, aquatic dispersal of the lowland species, *P. maculata,* might still have been possible within the lowland stream network sites on the west side of Upper Bukit Timah Road (Sites 1–3) until 1945, before development impacted this area. Dispersal by walking between streams within the higher order lowland stream network (which would presumably be more prone to flooding than the highland/hill streams) would be consistent with the observed intermediacy of the gene pool of the Site 2 population in Bukit Gombak among the sites to the west of Upper Bukit Timah Road (Sites 1–3) (Table 2 and Figure 3), which was not seen in the more terrestrial disperser *J. singaporensis*. Presently, however, walking across urban drainage systems by even the lowland‐dwelling *P. maculata* is unlikely, as the near vertical walls of concrete drains are too steep for crabs to scale (D. Ng, pers. obs.) and water quality differs considerably (e.g., higher temperature, pH, flow) from those of Singapore's natural forest streams in which freshwater crabs are restricted to (Chua et al., 2015; Yeo & Lim, 2011). In addition, further fine‐scale genetic structuring between the Bukit Gombak and Bukit Batok populations of *P. maculata* was observed, which is congruent with their isolation by another major road (now Hillview Avenue) by 1969, whereas Sites 1 and 2 within the Bukit Gombak forest were still not fully separated by major road constructions in 1969.

Habitat fragmentation and genetic isolation tend to create smaller populations that are more susceptible to forces of genetic drift, and thus often suffer from reduced genetic diversity (Dixo, Metzger, Morgante, & Zamudio, 2009; Keller & Largiadèr, 2003; Magle, Ruell, Antolin, & Crooks, 2010; Noël, Ouellet, Galois, & Lapointe, 2007; Vandergast et al., 2009). However, genetic diversity was similarly low in both species despite their vastly different population demographics (area of occupancy of *P. maculata* is more than ten‐fold larger): Only seven COI haplotypes were found in >90 individuals for each species across four populations, as such the number of haplotypes per sampled individuals is 3–9× lower than previous reports for other species of endemic freshwater crabs (Cook, Pringle, & Hughes, 2008; Daniels, 2011; Fang et al., 2015), and observed nucleotide diversity estimates were also comparably low with respect to other freshwater crab species (Cook et al., 2008; Daniels, 2011; Daniels, Stewart, & Cook, 2002; Fang et al., 2015). This suggests that evolutionary forces acting at a larger scale than individual population level may be responsible. One candidate is island isolation (Frankham, 1997) in general, and its effect on freshwater crab populations in Singapore in particular. Low genetic variability as was observed in the present study has been similarly documented across many island taxa largely due to genetic isolation from larger mainland populations, and subsequent inbreeding (Boff et al., 2014; Furlan et al., 2012; Jensen et al., 2013). In Singapore, this effect is likely to be exacerbated by the effects of habitat loss and fragmentation discussed above, and has been observed in other threatened forest‐dwelling species in Singapore such as primates and birds (Ang, Srivathsan, Md.‐Zain, Ismail, & Meier, 2012; Sadanandan & Rheindt, 2015). In addition, similar patterns of gradual and mild population contractions were estimated ~1500–2500 years ago for both *P. maculata* and *J. singaporensis* (Figure S5), and not a population expansion which usually follows after founding events. Inbreeding for individual populations was not detected in either species which suggests that any fine‐scale genetic isolation events were fairly recent, and that the populations are still genetically stable (i.e., no signs of inbreeding, or pressing evidence of low effective population size). Populations such as *J. singaporensis* at Bukit Timah and *P. maculata* at Bukit Batok, which had just one COI haplotype each, might even be the result of single dispersal (founding) events with no subsequent exchange between populations. Sampling of historical populations would help us to better understand if and when haplotypes might have been lost through bottleneck events; however, there is little published data (Klaus et al., 2013; Poettinger & Schubart, 2014) and those from Singapore were of a haplotype already detected in the present study (Figure 2), so we are unable to confirm if haplotype loss has occurred.

*Johora singaporensis* is one of the 100 most threatened species in the world (Baillie & Butcher, 2012). If it follows the trend of high extinction rates in island endemics, prolonged isolation of the existing small populations may eventually lead to inbreeding depression and population extinction (Frankham, 1998), even if suitable habitat conditions are maintained at each site. Current plans for urban development do not directly threaten the remaining populations of *J. singaporensis* because they are situated within a nature reserve (Bukit Timah, Site 4), a security area (Bukit Gombak, Site 1), and other areas designated as green spaces (Bukit Gombak and Bukit Batok, Sites 2 and 3) (Population White Paper, Ministry of National Development, 2013). However, the populations will remain isolated. The recovery potential of *J. singaporensis* is presumably high due to its relatively short generation time of 1.5 years and a fairly large brood size (100 eggs per brood) (D. Ng, pers. obs.), although its survivorship rates to maturity are unknown. There might also be yet undiscovered populations given that one population was recently discovered in the eastern part of Bukit Timah Nature Reserve (Li et al., 2015). Such positive developments, however, should not distract from the fact that additional conservation efforts are necessary given that there is often a temporal lag between extinction and losses of ecosystem functioning as a result of habitat fragmentation (Haddad et al., 2015). Hence, regular population monitoring of the wild populations should be implemented to allow for early detection of any signs of inbreeding depression, such as reduced clutch size. Current conservation efforts for *J. singaporensis* include captive breeding, translocation, improving habitat suitability, and population monitoring (Ng, McGowan, et al., 2015). Even though some species appear to thrive despite low genetic variability (e.g., Milot, Weimerskirch, Duchesne, & Bernatchez, 2007), the risk of reduced fitness through inbreeding should not be dismissed, especially given the low genetic variability within, and strong genetic differences between the wild populations. Hence, we recommend continued captive breeding that mixes reproductive individuals from different streams, and if successful, is followed by subsequent translocation to suitable hill streams in protected areas currently not inhabited by *J. singaporensis*. “Natural” genetic rescue (Ingvarsson, 2001) of the wild populations may also be implemented perhaps by creating corridors suitable for dispersal, in order to increase their capacity for survival, especially in this era of rapid global climatic change. Such dispersal corridors may also help conserve other locally threatened species (e.g., the secondary freshwater crab *Geosesarma nemesis*, and the frog *Megophrys nasuta*) that are associated with the same habitats. All this work should be preceded by detailed surveys of flora, fauna, and the hydrological conditions in the affected habitats.

It appears likely that the subtle differences between the two crab species are likely due to the short time frame and the fast development of Singapore over the last two centuries (Turnbull, 1989). These changes could not have been detected using just mitochondrial markers, but required thousands of genome‐wide loci mined by ddRAD‐Seq (Peterson, Weber, Kay, Fisher, & Hoekstra, 2012). As DNA sequencing costs decrease and the RADseq method of sampling SNPs grows in popularity (reviewed in Andrews, Good, Miller, Luikart, & Hohenlohe, 2016), we nevertheless recommend that studies adopt a layered approach. A first assessment can be performed based on sets of homologous loci such as mitochondrial markers (e.g., COI) which can yield initial genetic diversity estimates and species confirmation for those species where juveniles, males, and females are morphologically disparate. Such confirmation is especially important for species complexes with suspected cryptic species (Ewers & Wares, 2012; Huang, Chen, Wei, Bu, & Wu, 2017; James et al., 2010; Yang et al., 2015). COI is particularly suitable for this purpose because it has broad taxon coverage due to its use in DNA barcoding and phylogeography (Crandall et al., 2008; Herbert, Cywinska, Ball, & deWaard, 2003; Tay et al., 2016). Moreover, data for large numbers of specimens can be readily obtained using multiplexed high‐throughput sequencing DNA barcoding methods (Meier, Wong, Srivathsan, & Foo, 2016). Deeper population genomic analyses can then be performed using the large genome‐wide SNP datasets on specimens identified as suitable for the population‐level analyses.

Here, a combination of techniques allowed us to find a surprising pattern of shared population genomic history of two freshwater crab species despite vastly different population demographics between them. Our findings of low genetic diversity provide crucial information for current captive breeding efforts of one of the 100 most threatened species in the world (Baillie & Butcher, 2012), and also insights into possible conservation actions for threatened populations in the wild.

## CONFLICT OF INTEREST

The authors declare no competing interests.

## AUTHOR CONTRIBUTIONS

Y.C.T., D.J.J.N., R.M., and D.C.J.Y. designed the study. Funding and laboratory support were provided by D.J.J.N., D.C.J.Y., R.M. and D.H. D.J.J.N. and Y.C. obtained permits and performed the field sampling. Y.C.T., D.J.J.N., and J.B.L. performed the laboratory work and collected genetic/genomic data. Y.C.T., D.J.J.N., R.M., D.C.J.Y., and D.H. were engaged in the genetic/genomic analyses and interpretation. Y.C.T. and D.J.J.N. wrote the manuscript. All authors edited and approved the final version of the article.

## DATA ACCESSIBILITY

Demultiplexed, raw Illumina sequencing ddRAD‐Seq data for each sample are available on Dryad (https://doi.org/10.5061/dryad.h4j7hv4). COI sequences are available on Genbank (Accession numbers MG010138–MG010329). Additional information is available in the Supplementary material.
