# Supplementary material for: Roads to isolation: Similar genomic history patterns in two species of freshwater crabs with contrasting environmental tolerances and range sizes
Source: Ecol Evol. 2018 Apr 15;8(9):4657–68. doi: 10.1002/ece3.4017 (PMC5938456; doi:10.1002/ece3.4017)
Supplement: Supplementary file 1 [file ECE3-8-4657-s001.pdf]

Table S1. Barcode, index assignments and sampling localities for (a) *J. singaporensis* and (b) *P. maculata* individuals used in RADseq analyses

	(a) *Johora singaporensis*						(b) *Parathelphusa maculata*					
Adapter tag	Illumina Index	Sample_filename	Sampling locality	Illumina Index	Sample_filename	Sampling locality	Illumina Index	Sample_filename	Sampling locality	Illumina Index	Sample_filename	Sampling locality
TGCAT		JSIN_BTM01.fq			JSIN_BGK101.fq			PMAC_BTM01.fq			PMAC_BGK101.fq	
CAACC		JSIN_BTM03.fq			JSIN_BGK102.fq			PMAC_BTM02.fq			PMAC_BGK102.fq	
GGTTG		JSIN_BTM04.fq			JSIN_BGK103.fq			PMAC_BTM03.fq			PMAC_BGK103.fq	
AAGGA		JSIN_BTM05.fq			JSIN_BGK104.fq			PMAC_BTM04.fq			PMAC_BGK104.fq	
AGCTA		JSIN_BTM06.fq			JSIN_BGK105.fq			PMAC_BTM05.fq			PMAC_BGK105.fq	
ACACA		JSIN_BTM07.fq			JSIN_BGK106.fq			PMAC_BTM07.fq			PMAC_BGK106.fq	
ACTTC		JSIN_BTM08.fq			JSIN_BGK107.fq			PMAC_BTM08.fq			PMAC_BGK108.fq	
ATGAG		JSIN_BTM09.fq			JSIN_BGK108.fq			PMAC_BTM09.fq			PMAC_BGK109.fq	
CGTAC		JSIN_BTM10.fq			JSIN_BGK109.fq			PMAC_BTM10.fq			PMAC_BGK110.fq	
CTGTC		JSIN_BTM11.fq			JSIN_BGK110.fq			PMAC_BTM11.fq			PMAC_BGK111.fq	
CTTGG		JSIN_BTM12.fq			JSIN_BGK111.fq			PMAC_BTM12.fq			PMAC_BGK112.fq	
GAGAT	1	JSIN_BTM13.fq	Bukit Tmah, Site 4	6	JSIN_BGK112.fq	Bukit Gombak, Site 1	1	PMAC_BTM13.fq	Bukit Tmah, Site 4	6	PMAC_BGK113.fq	Bukit Gombak, Site 1
GCTGA		JSIN_BTM14.fq			JSIN_BGK113.fq			PMAC_BTM14.fq			PMAC_BGK114.fq	
GTAGT		JSIN_BTM15.fq			JSIN_BGK114.fq			PMAC_BTM15.fq			PMAC_BGK115.fq	
GTCCG		JSIN_BTM16.fq			JSIN_BGK115.fq			PMAC_BTM16.fq			PMAC_BGK116.fq	
GCATG		JSIN_BTM18.fq			JSIN_BGK118.fq			PMAC_BTM17.fq			PMAC_BGK117.fq	
AACCA		JSIN_BTM19.fq			JSIN_BGK119.fq			PMAC_BTM18.fq			PMAC_BGK118.fq	
CGATC		JSIN_BTM20.fq			JSIN_BGK120.fq			PMAC_BTM19.fq			PMAC_BGK119.fq	
TCGAT		JSIN_BTM21.fq			JSIN_BGK121.fq			PMAC_BTM20.fq			PMAC_BGK120.fq	
ACTGG		JSIN_BTM22.fq			JSIN_BGK122.fq			PMAC_BTM21.fq			PMAC_BGK121.fq	
ATACG		JSIN_BTM23.fq			JSIN_BGK123.fq			PMAC_BTM22.fq			PMAC_BGK122.fq	
ATTAC		JSIN_BTM24.fq			JSIN_BGK124.fq			PMAC_BTM23.fq			PMAC_BGK125.fq	
CATAT		JSIN_BTM25.fq			JSIN_BGK125.fq			PMAC_BTM24.fq			PMAC_BGK126.fq	
CGGCT		JSIN_BBK01.fq			JSIN_BGK201.fq			PMAC_BBK01.fq			PMAC_BGK201.fq	
GACAC		JSIN_BBK02.fq			JSIN_BGK202.fq			PMAC_BBK02.fq			PMAC_BGK202.fq	
GCCGT		JSIN_BBK03.fq			JSIN_BGK203.fq			PMAC_BBK03.fq			PMAC_BGK203.fq	
TAGTA		JSIN_BBK04.fq			JSIN_BGK204.fq			PMAC_BBK04.fq			PMAC_BGK204.fq	
AATTA		JSIN_BBK05.fq			JSIN_BGK205.fq			PMAC_BBK05.fq			PMAC_BGK205.fq	
ACGGT		JSIN_BBK06.fq			JSIN_BGK206.fq			PMAC_BBK06.fq			PMAC_BGK206.fq	
CGAAT		JSIN_BBK07.fq			JSIN_BGK207.fq			PMAC_BBK07.fq			PMAC_BGK207.fq	
CGGTA		JSIN_BBK08.fq			JSIN_BGK209.fq			PMAC_BBK09.fq			PMAC_BGK208.fq	
CGTCG		JSIN_BBK09.fq			JSIN_BGK210.fq			PMAC_BBK10.fq			PMAC_BGK209.fq	
GAGTC		JSIN_BBK10.fq			JSIN_BGK211.fq			PMAC_BBK12.fq			PMAC_BGK210.fq	
GGCCA		JSIN_BBK11.fq			JSIN_BGK212.fq			PMAC_BBK14.fq			PMAC_BGK212.fq	
TACCG	5	JSIN_BBK12.fq	Bukit Batok, Site 3	7	JSIN_BGK213.fq	Bukit Gombak, Site 2	5	PMAC_BBK15.fq	Bukit Batok, Site 3	7	PMAC_BGK213.fq	Bukit Gombak, Site 2
TATAC		JSIN_BBK13.fq			JSIN_BGK214.fq			PMAC_BBK16.fq			PMAC_BGK214.fq	
TTACC		JSIN_BBK14.fq			JSIN_BGK215.fq			PMAC_BBK18.fq			PMAC_BGK215.fq	
CTGAT		JSIN_BBK15.fq			JSIN_BGK216.fq			PMAC_BBK23.fq			PMAC_BGK216.fq	
GGATA		JSIN_BBK16.fq			JSIN_BGK217.fq			PMAC_BBK24.fq			PMAC_BGK217.fq	
GTCGA		JSIN_BBK17.fq			JSIN_BGK218.fq			PMAC_BBK26.fq			PMAC_BGK218.fq	
TACGT		JSIN_BBK18.fq			JSIN_BGK219.fq			PMAC_BBK27.fq			PMAC_BGK219.fq	
TCACG		JSIN_BBK19.fq			JSIN_BGK220.fq			PMAC_BBK28.fq			PMAC_BGK220.fq	
TCTGC		JSIN_BBK20.fq			JSIN_BGK221.fq			PMAC_BBK29.fq			PMAC_BGK221.fq	
TGGAA		JSIN_BBK21.fq			JSIN_BGK222.fq			PMAC_BBK30.fq			PMAC_BGK222.fq	
CTGCG		JSIN_BBK22.fq			JSIN_BGK223.fq			PMAC_BBK31.fq			PMAC_BGK224.fq	
GGCTC		JSIN_BBK23.fq			JSIN_BGK224.fq			PMAC_BBK32.fq			PMAC_BGK225.fq	
TCAGT		JSIN_BBK24.fq			JSIN_BGK225.fq			PMAC_BBK33.fq			PMAC_BGK226.fq	

(a)

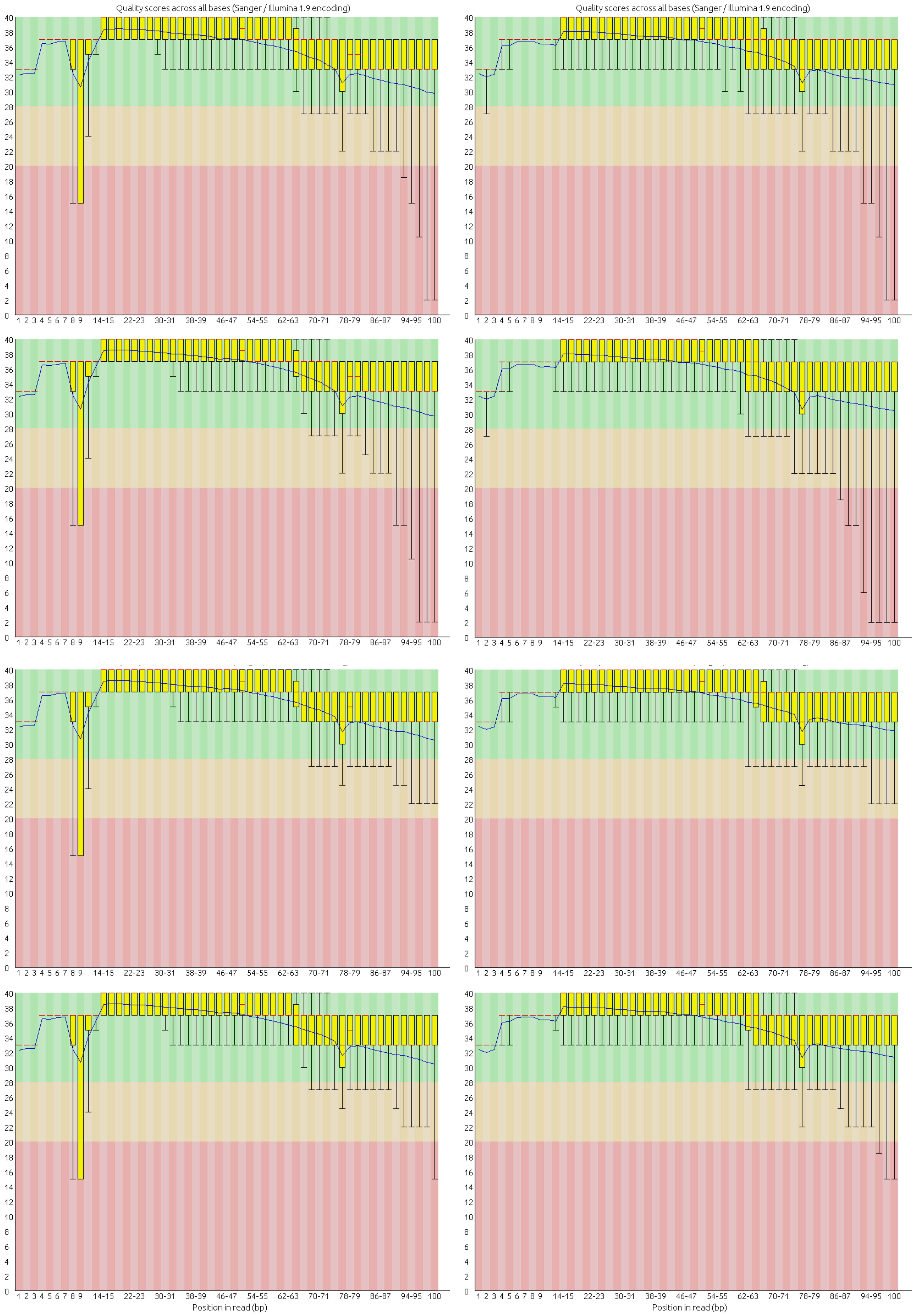

Quality scores across all bases (Sanger / Illumina 1.9 encoding)
Quality scores across all bases (Sanger / Illumina 1.9 encoding)
Position in read (bp)
Position in read (bp)

(b)

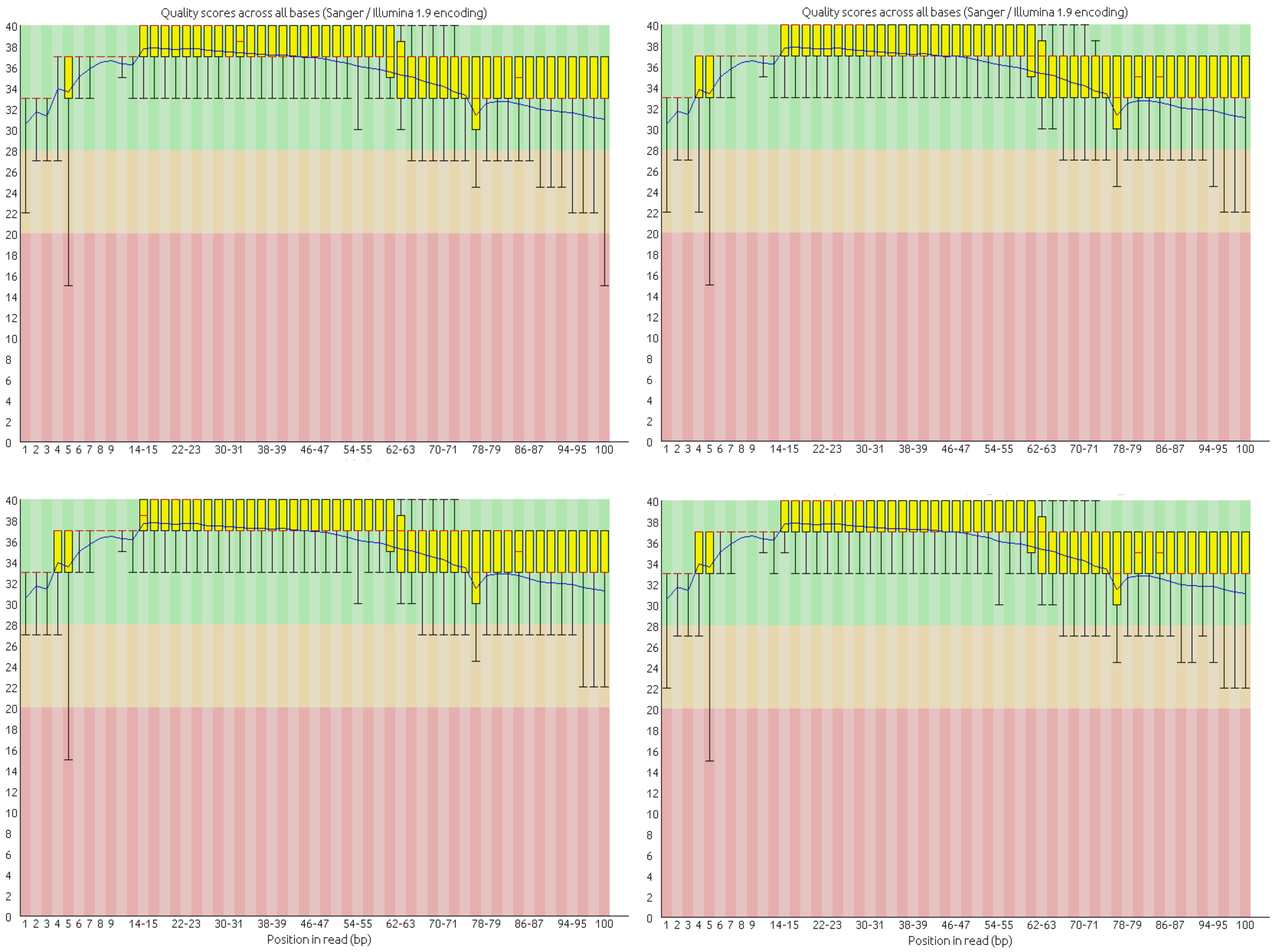

Figure S1. Quality scores across reads for data from Read 1 of each of the Illumina HiSeq libraries (a) *J. singaporensis*, (b) *P. maculata*.

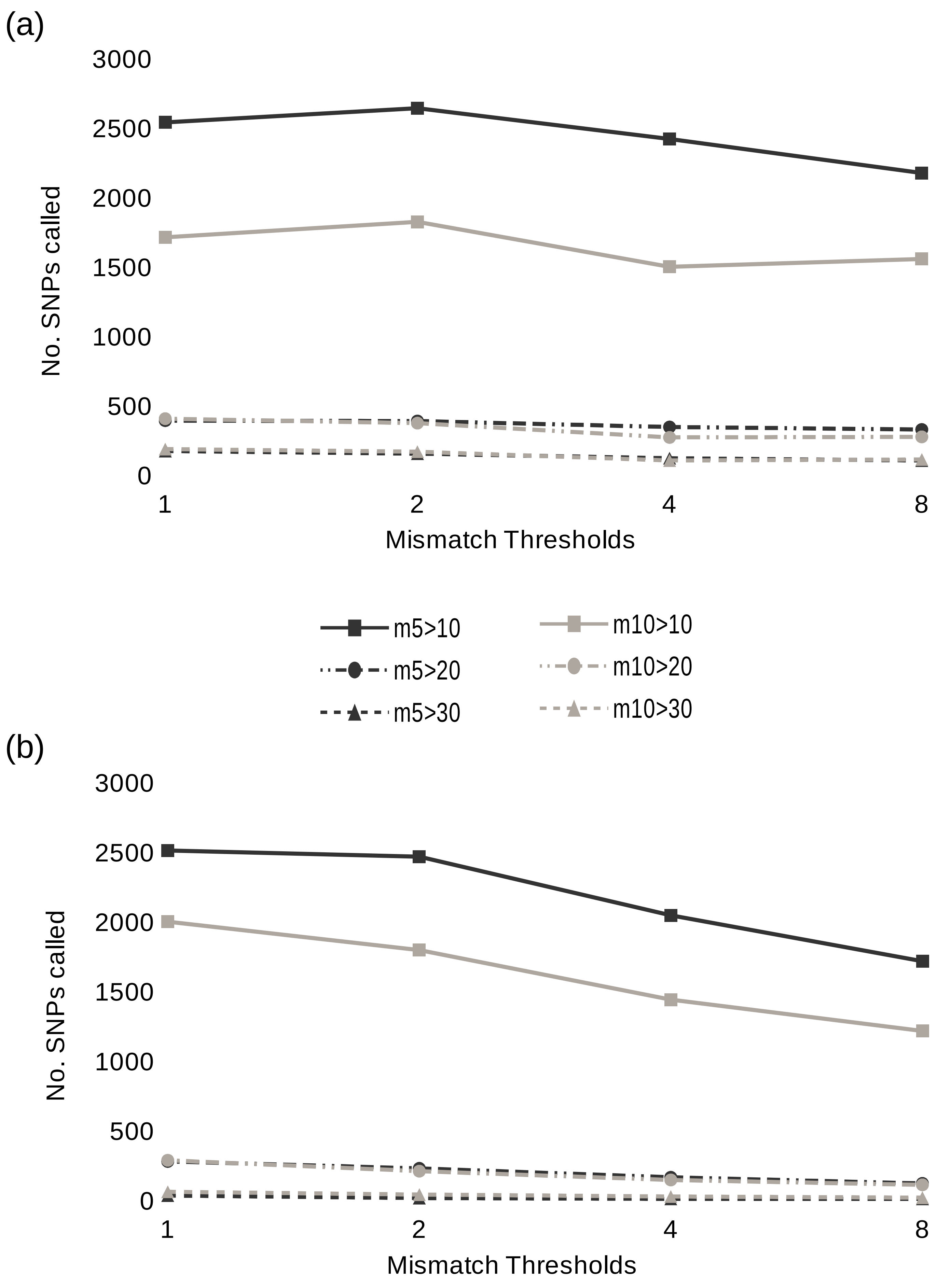

Figure S2. Number of SNPs called across datasets produced by different parameter settings for (a) *J. singaporensis* and (b) *P. maculata.*

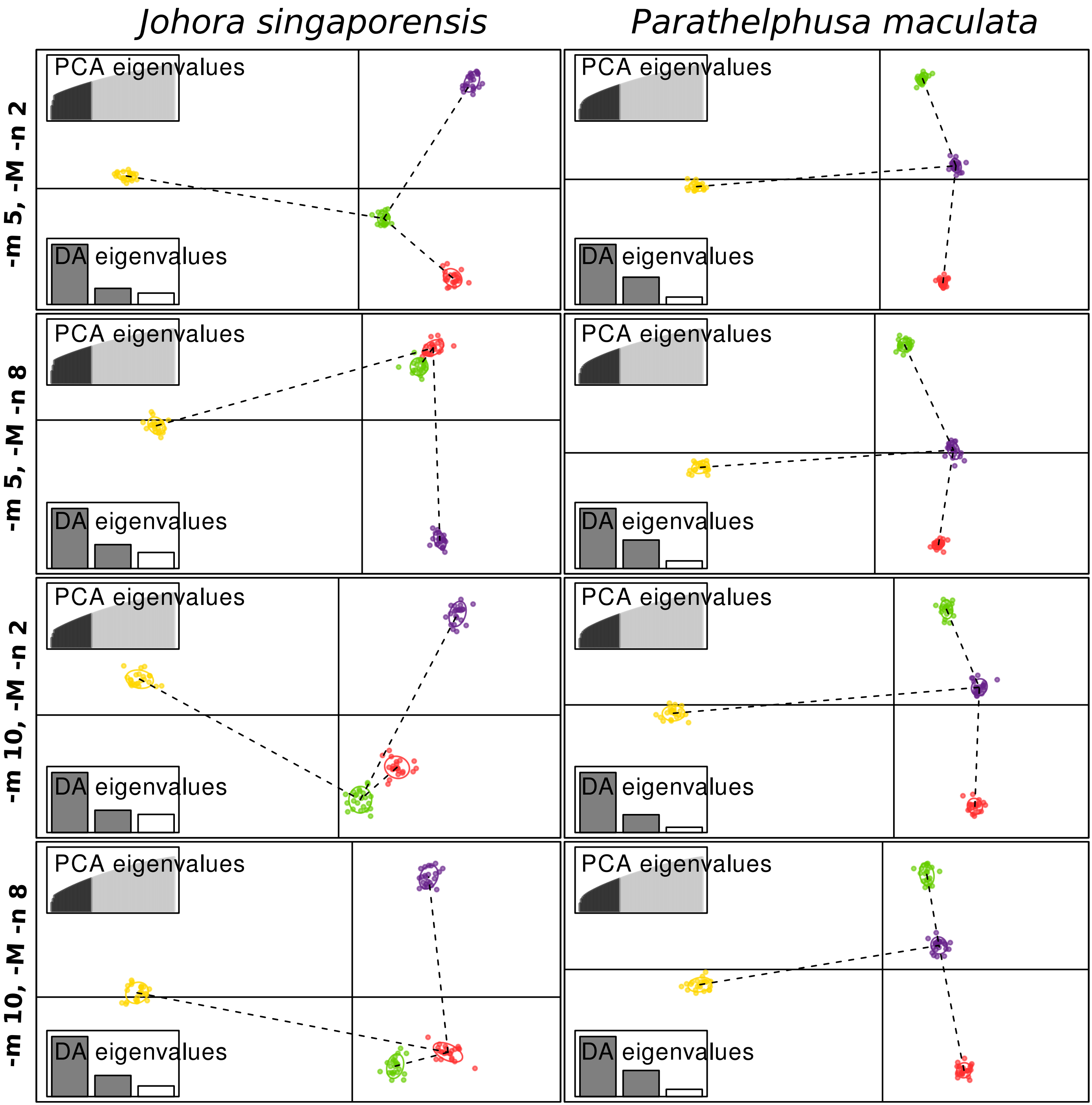

Figure S3. Sensitivity analysis of effects of STACKS parameter settings via DAPC plots for (left) *J. singaporensis* and (right) *P. maculata*. Samples are colour-coded following Figure 2.

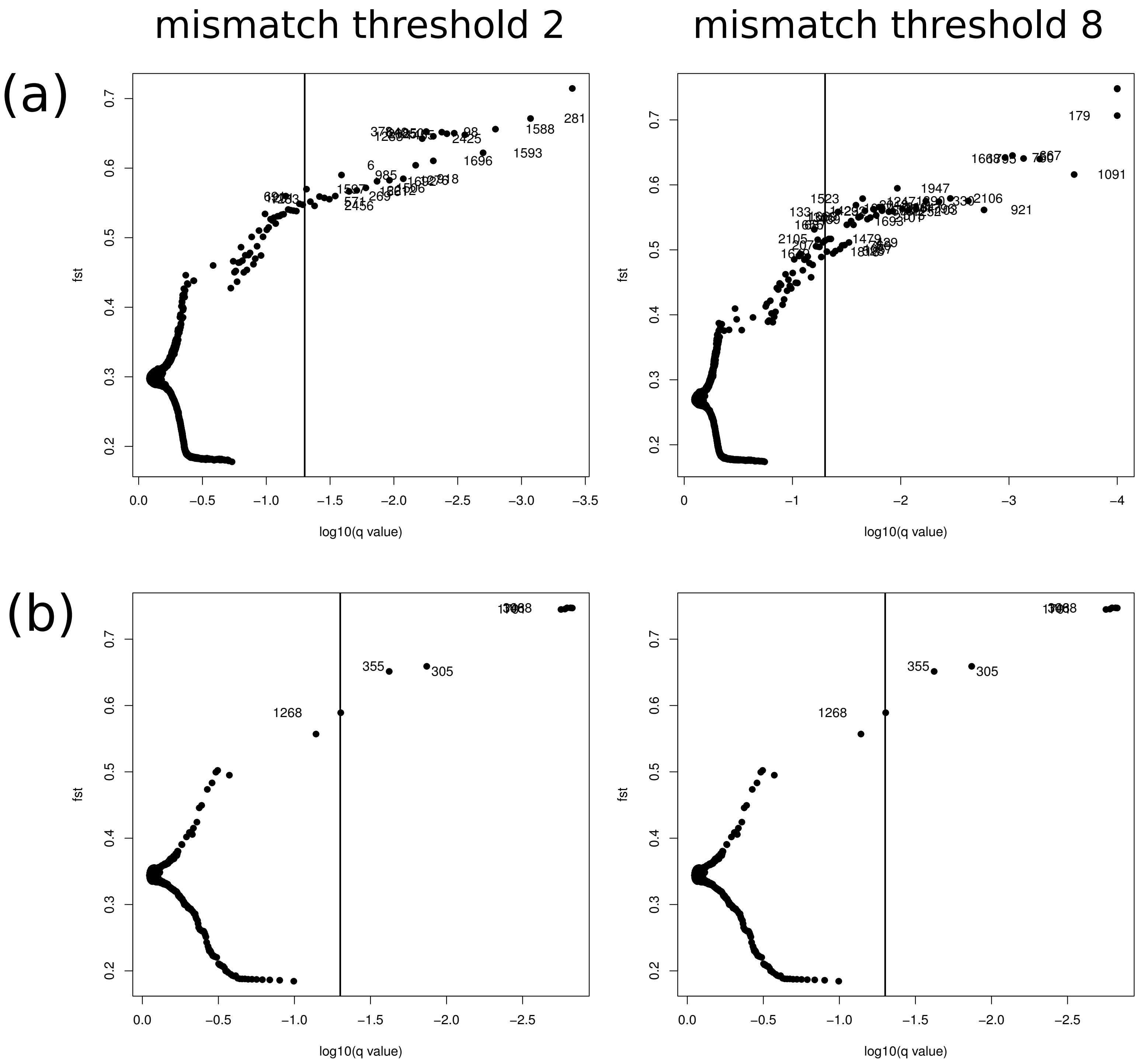

Figure S4. Bayescan analyses of SNP loci from representative runs of (a) *J. singaporensis* and (b) *P. maculata* at mismatch thresholds (-m) of 2 and 8, and final locus depth of 10. The vertical lines represent the cutoff at FDR of 0.05.

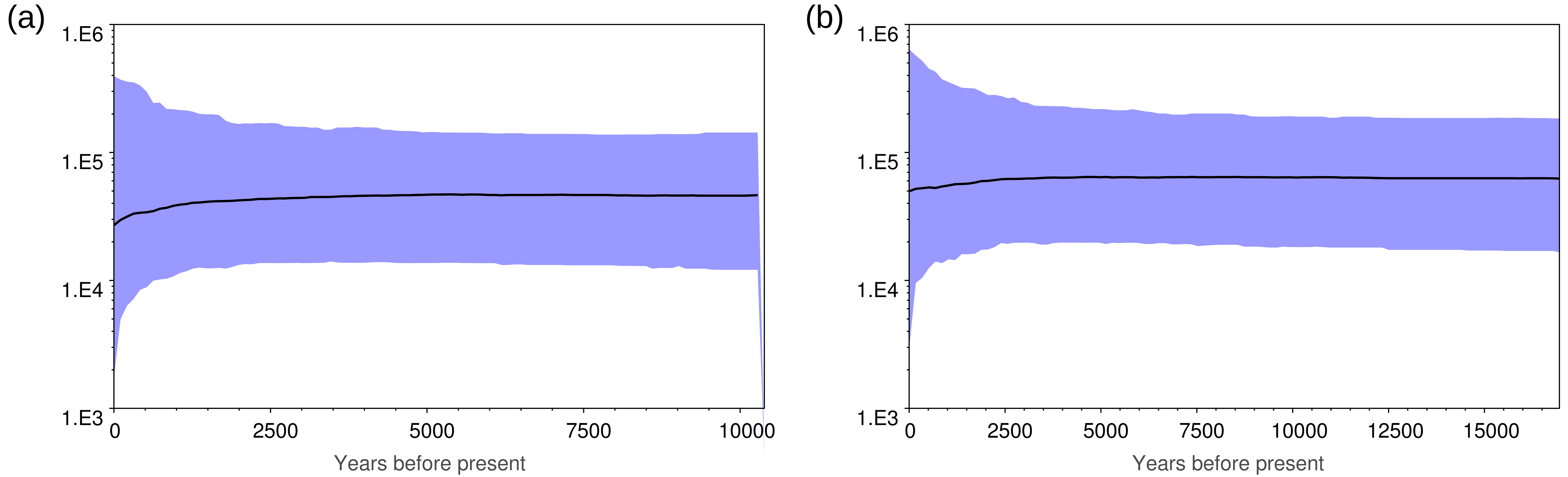

Figure S5. Bayesian Skyline Plots for (a) *J. singaporensis* and (b) *P. maculata*.

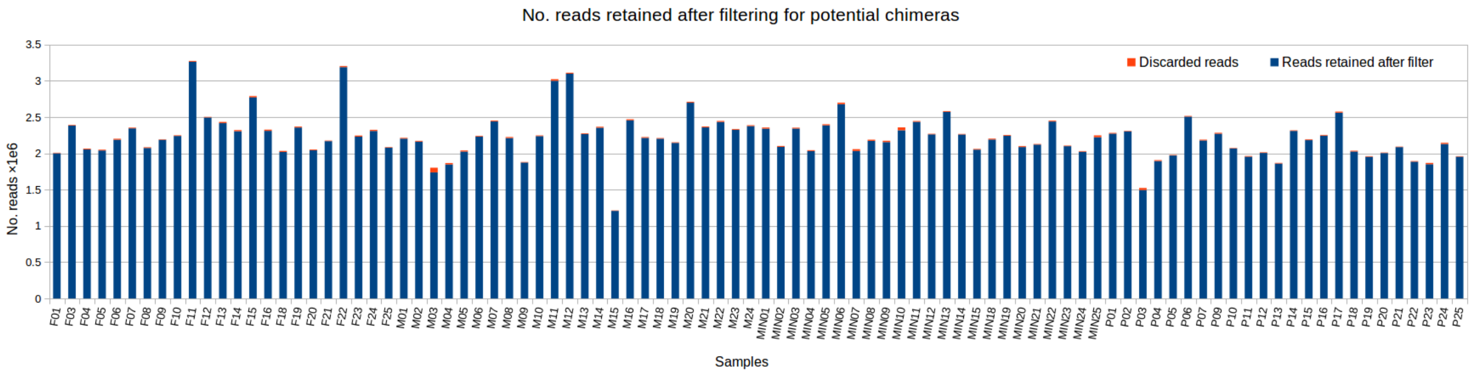

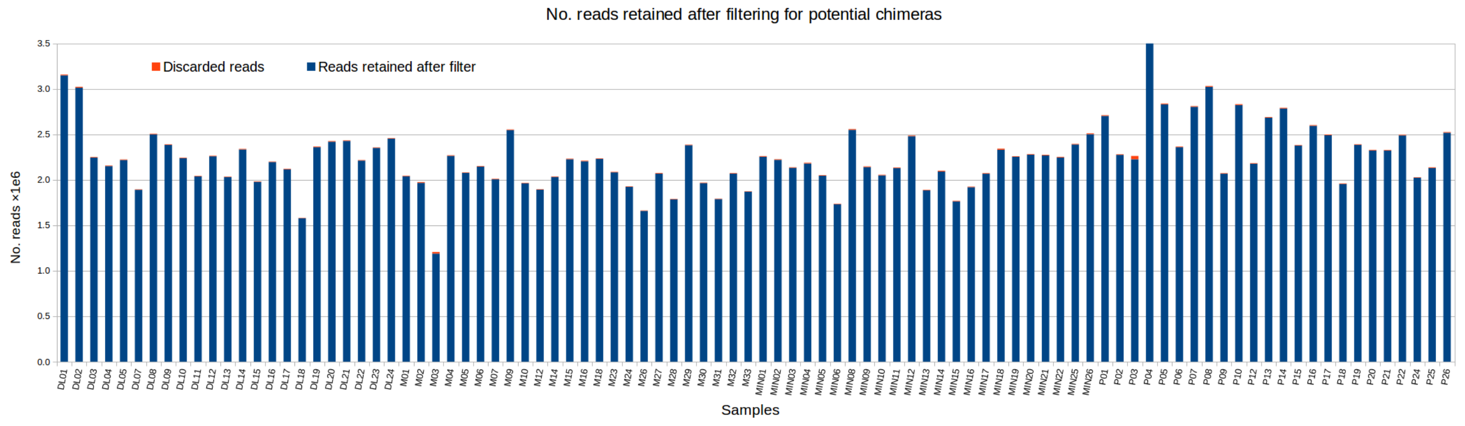

Figure S6. Read counts before and after filtering for potential chimeric sequences for (a) *J. singaporensis* and (b) *P. maculata*.

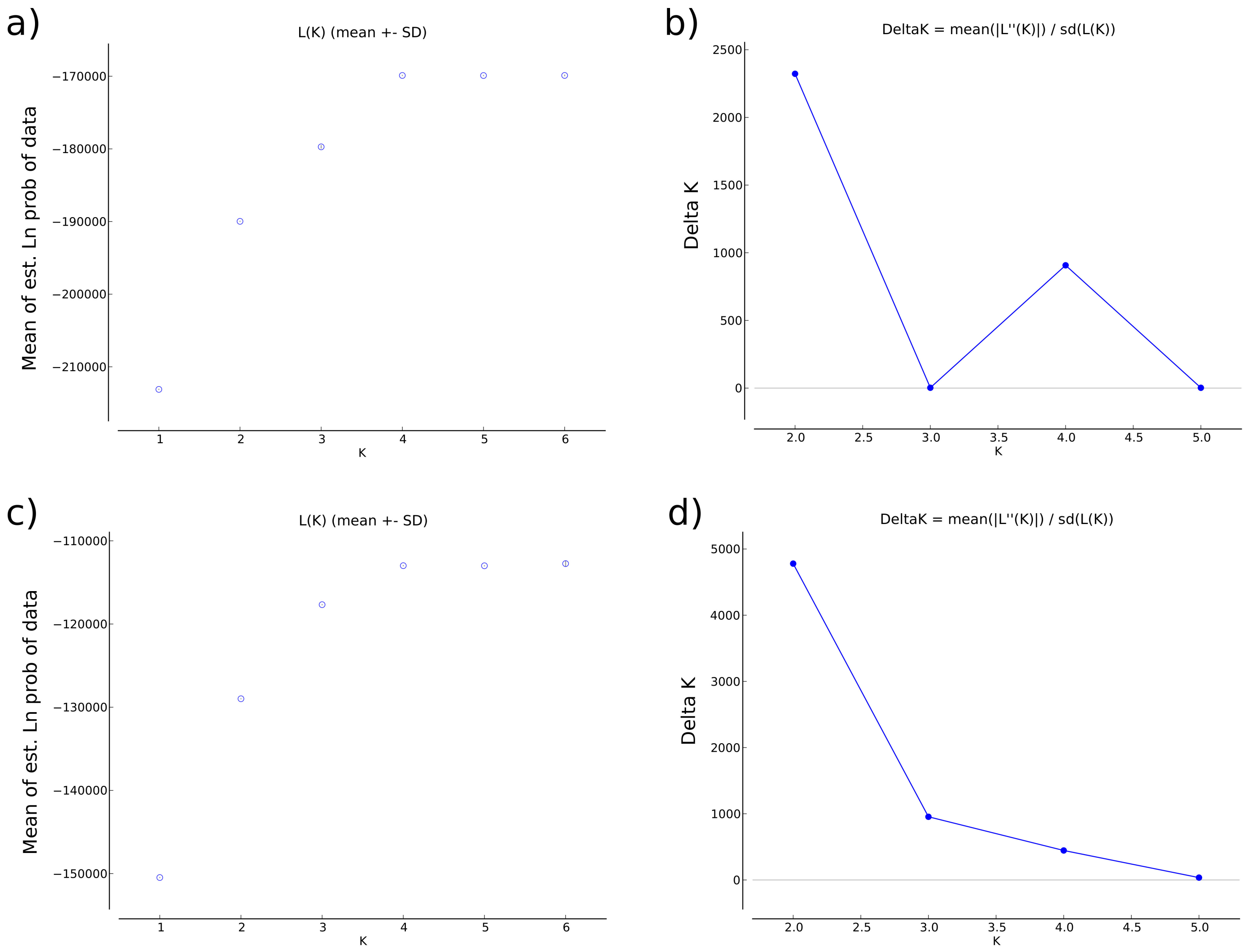

Figure S7. STRUCTURE HARVESTER analyses based on 2,617 and 2,470 SNP loci harvested under a mismatch threshold of M=2, final 'stack' depth of 10 and MAF>0.015, for K=1–6 over ten iterations for (a,b) *J. singaporensis* and (c,d) *P. maculata*. (a,c) Plots of mean likelihood L(K) and variance per K, (b,d) plots of changes in probabilities of K.
